# An in vitro culture platform for studying the effect of collective cell migration on spatial self-organization within induced pluripotent stem cell colonies

**DOI:** 10.1186/s13036-023-00341-z

**Published:** 2023-03-30

**Authors:** Mee-Hae Kim, Masaki Kuroda, Ding Ke, Naruchit Thanuthanakhun, Masahiro Kino-oka

**Affiliations:** 1 Department of Biotechnology, Graduate School of Engineering, Osaka University, 2-1 Yamadaoka, Suita, Osaka 565-0871 Japan; 2 Research Base for Cell Manufacturability, Osaka University, 2-1 Yamadaoka, Suita, Osaka 565-0871 Japan

**Keywords:** Human induced pluripotent stem cells, Ring culture system, Actin actomyosin, Collective cell migration, Epithelial mesenchymal transition, Mesendoderm lineage segregation

## Abstract

**Background:**

Human induced pluripotent stem cells (hiPSCs) provide an in vitro system to identify the impact of cell behavior on the earliest stages of cell fate specification during human development. Here, we developed an hiPSC-based model to study the effect of collective cell migration in meso–endodermal lineage segregation and cell fate decisions through the control of space confinement using a detachable ring culture system.

**Results:**

The actomyosin organization of cells at the edge of undifferentiated colonies formed in a ring barrier differed from that of the cells in the center of the colony. In addition, even in the absence of exogenous supplements, ectoderm, mesoderm, endoderm, and extraembryonic cells differentiated following the induction of collective cell migration at the colony edge by removing the ring-barrier. However, when collective cell migration was inhibited by blocking E-cadherin function, this fate decision within an hiPSC colony was altered to an ectodermal fate. Furthermore, the induction of collective cell migration at the colony edge using an endodermal induction media enhanced endodermal differentiation efficiency in association with cadherin switching, which is involved in the epithelial-mesenchymal transition.

**Conclusions:**

Our findings suggest that collective cell migration can be an effective way to drive the segregation of mesoderm and endoderm lineages, and cell fate decisions of hiPSCs.

**Supplementary Information:**

The online version contains supplementary material available at 10.1186/s13036-023-00341-z.

## Highlights

• Ring culture system induces distinct changes in actomyosin organization at the iPSC colony edge.

• Removal of ring culture system induces collective cell migration at the iPSC colony edge.

• Collective cell migration modulates self-organized fate patterning decisions in iPSC-derived gastrulation-stage meso–endoderm.

• Cadherin switching during collective cell migration enhances endodermal differentiation.

**Supplementary Information:**

The online version contains supplementary material available at 10.1186/s13036-023-00341-z.

## Introduction

Human pluripotent stem cells (hPSCs), including embryonic stem cells (ESCs) and induced pluripotent stem cells (iPSCs), are widely used in regenerative medicine and experimental models for studying development and diseases because of their pluripotent potential to differentiate into all cell lineages: endoderm, ectoderm, and mesoderm germ layers [1–3]. The use of hPSCs provides great opportunities to model and study embryonic and human development.

Recent advances in directed differentiation protocols for hPSCs in combination with soluble morphogens and growth factors have facilitated the generation of various hPSC-derived in vitro models of embryonic development and associated diseases [4–8]. The use of micropatterning tools has also led to the highly efficient and reproducible generation of “hPSC-derived gastruloids,” where spatial organization during embryonic gastrulation can be mimicked in vitro, which allows the study of the roles of the stem cell niche during the early and late stages of differentiation events [5, 6]. In addition to soluble morphogens and growth factors, hPSC micropattern colonies differentiate in radially symmetrical patterns of ectoderm, mesoderm, endoderm, and extra embryonic cells from the center to the edge [9–11]. hPSCs differentiate into meso–endodermal cells and undergo collective cell migration and epithelial-mesenchymal transition (EMT) through activation of the activin/nodal, BMP, FGF, and Wnt/β-catenin signaling pathways [6, 9–11]. Moreover, meso–endodermal cells undergo EMT, closely paralleling the organization of the primitive streak [12]. However, creating a culture platform with spatiotemporal control of cell behavior to study signaling dynamics during development remains challenging.

An understanding of morphogenetic processes and their responses to guidance cues provides details of the morphogenic events that occur during embryonic development [13–18]. Two major morphogenetic events, EMT and collective cell migration, clearly highlight the role of mechano-transduction in regulating cellular behavior during development [12–14]. The cadherin switch from E-cadherin to N-cadherin is a prime indicator of cells undergoing EMT and is crucial for the specification of the primitive streak and other embryogenesis events [13, 14, 19, 20]. These changes are associated with the acquisition of migratory behavior, suggesting that the actin cytoskeleton regulates the mechanical behavior of cells [15–22]. Importantly, cell fate is governed by a complex signaling network coupled with mechanical cues, which ultimately leads to self-organization to form spatial patterns during development [12, 13]. Therefore, most in vitro systems focus on the extrinsic physical forces associated with dynamic cell behaviors that direct subsequent fate decisions of a cell, for assessing spatial self-organization during development [13, 17, 18].

In this study, we hypothesize that collective cell migration modulates self-organized fate patterning decisions in human iPSC (hiPSC)-derived gastrulation-stage meso–endoderm. To test this hypothesis, we investigated the dynamics of spatial self-organization within hiPSC colonies using a detachable ring culture system during differentiation, rather than culturing pluripotent colonies on circular domains before differentiation. This is a polydimethylsiloxane (PDMS)-based culture system having a magnetic ring with a biocompatible chamber containing the thinnest transparent cell culture layer available, resulting in high optical quality. Furthermore, this culture system can be used as a physical barrier to build physically confined spaces; in contrast, the removal of the ring can trigger cell migration. Accordingly, we investigated the response of the actomyosin cytoskeleton to confined environments and its alteration during cell migration after the removal of the physical barrier of the ring culture system. This culture platform provides a simple method to study the role of collective cell migration behavior in spatial self-organization within a hiPSC colony and discusses the fundamental mechanisms of cell fate patterning decisions with respect to EMT.

## Results

### Design and characterization of a ring culture system

**Fig. 1:**
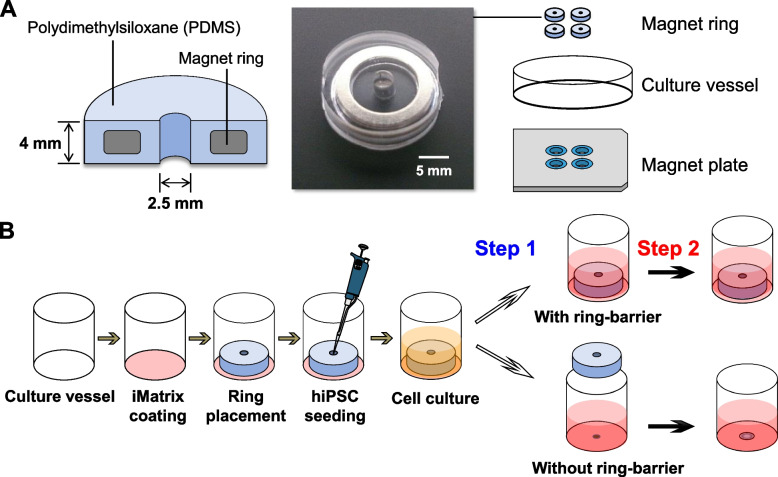
Establishment of ring culture system and experimental design. **A** Ring fabrication diagram showing the structure of PDMS-based culture system embedded with ring magnet. **B** Experimental design for hiPSC colony formation (Step 1) and self-organization of spatial patterns (Step 2) within the hiPSC colony with or without ring-barrier. The barrier made from detachable ring culture system is placed on culture surface and cells are seeded within this area; upon confluency, the barrier is removed, which results in a center and edge of the colony, a cell-free space that allows cell migration. In Step 2, the cell migration was monitored and the spatial self-organization within the hiPSC colonies was compared under culture conditions with and without HA exposure

We present a newly developed and simple in vitro culture platform that can be applied to investigate the effect of the migratory behavior of cells on the spatial self-organization of hiPSCs. Figure 1 depicts the structure of the PDMS-based culture system embedded with the ring magnet, used in this study. The proposed PDMS culture system is constructed based on a magnetic ring embedded in a resin with high gas permeability. A magnetic ring embedded in PDMS was fabricated using a standard soft lithography technique. To facilitate easy removal of the ring from the culture surface, this culture system consists of two complementary parts composed of a simple removable PDMS ring with an embedded magnetic ring and a stainless-steel plate, allowing the attachment and detachment of two parts repeatedly. The ring is composed of a closed culture vessel with a height and internal diameter of 1.3 and 2.5 mm, respectively. This method allows single colony formation from multiple cells under space restriction and can induce cell migration by a simple method using rings that are placed before seeding cells and removed after cell colony formation.

### Characterization of single colony formation by hiPSC population in a ring culture system

**Fig. 2:**
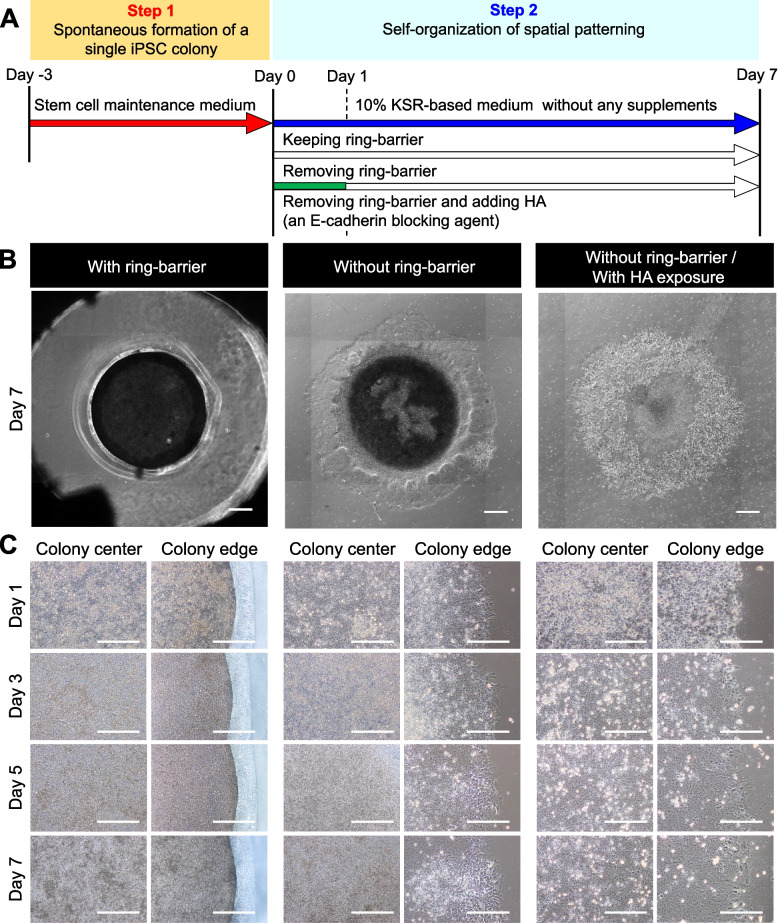
Spatial self-organization within the hiPSC colony with or without the ring-barrier. **A** Experimental design for hiPSC colony formation (Step 1) and self-organization of spatial patterns (Step 2) within the hiPSC colony with or without the ring-barrier. The barrier made from detachable ring culture system is placed on culture surface and cells are seeded within this area; upon confluency, the barrier is removed, which results in a center and edge of the colony, a cell-free space that allows cell migration. In Step 2, the cell migration was monitored and the spatial self-organization within the hiPSC colonies was compared under culture conditions with and without HA exposure. **B** Representative morphologies at different time points during spatial self-organization within hiPSC colony with or without ring-barrier are shown. The hiPSC colonies were compared after further exposure to HA in culture conditions without the ring-barrier. Colony diameter, 2.5 mm. Scale bar, 500 μm. **C** Representative images for cell morphology at the center and edge of the hiPSC colony in the image (**B**). All experiments were repeated independently at least three times with similar results

To study the effect of physical barriers on hiPSC colony formation by cell populations, we present a method that utilizes a PDMS-based ring culture system to spatially restrict cell adhesion to the underlying substrate (Fig. 2A). A magnetic ring was placed on the iMatrix-coated culture surface, and cells were seeded and cultured under culture conditions to maintain pluripotency. After seeding the cultures at a high density, the cells grew rapidly to generate multiple single cell-derived colonies of 2.5 mm diameter. Within a colony, cells at the edge grew faster than those at the center and had a higher local density around this barrier in keeping the barrier made from the ring culture system on day 10 (Fig. 2B, C). The cells fully grew as a tightly packed colony and well-defined edge in a confined space of the ring culture system. The edge of the colony showed a multilayered appearance along the sidewall of the ring culture system in a closed space.

To examine the effect of collective cell migration at the edge of the spatially confined hiPSC colony, the ring culture system was removed on day 0 and cultured in a KSR-based medium without the addition of exogenous supplements to study the effect of collective cell migration alone. From days 1 to 5, cells at the colony edge flattened and started to migrate away from the colony (Fig. 2B, C). By day 7, the migrating cells presented flattened, elongated, and fibroblastic morphology. In contrast, cells in the central region of the original colony remained highly proliferative and retained epithelial morphology. Multilayer structures were formed at the boundary between the inside and outside of the ring-barrier.

The spatial pattern of cells within the hiPSC colony highly depends on cell–cell interactions. Since E-cadherin is known to be linked to the actin cytoskeleton, cell–cell adhesion further enhances collective cell migration [23–25]. To further confirm the roles of collective cell migration on the self-organization of cells within the hiPSC colony, botulinum hemagglutinin (HA), an E-cadherin adhesion-blocking agent was added to hiPSC colony [26, 27]. On day 1 after HA exposure for 24 h, the contact between the cells was lost and some cells appeared highly rounded up (Fig. 2B, C). However, it was completely restored by day 7. The cells at the colony edge showed a flat and elongated fibroblast morphology. Taken together, these results suggest that removal of the ring barrier is sufficient to induce self-organization of hiPSCs through collective cell migration at the colony edge, which suggests that stable adherens junctions during collective cell migration are necessary for self-organization of spatial patterning hiPSC colony.

### Characterization of self-organization of spatial patterning hiPSCs with or without ring-barrier in the absence of exogenous differentiation factors

**Fig. 3:**
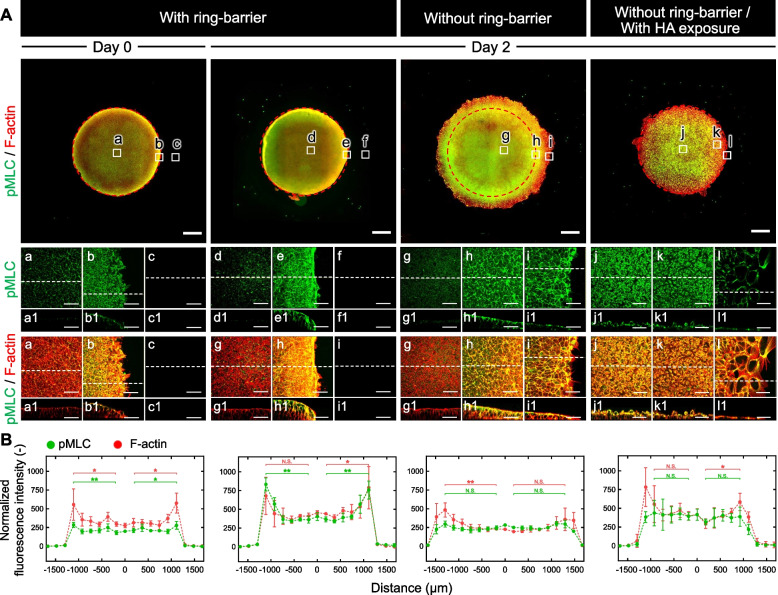
Characterization of spatial self-organization in the hiPSC colony with and without the ring-barrier. **A** Representative images in the *XY* plane showing pMLC and F-actin within the hiPSC colony in cultures with (a–c) the ring-barrier at day 0, and with (d–f) or without (g–i) the ring-barrier at day 2. The hiPSC colonies were compared after further exposure to HA in culture conditions without the ring barrier (j–l) at day 2. Images show the whole colony. Scale bar, 500 μm. Panels a1–l1 are the tomograms sectioned at the *XZ* plane (yellow dashed lines) in panels a-l. Red dashed lines indicate location of the ring culture system. Scale bar, 50 μm. **B** Quantification of staining intensity of pMLC and F-actin signals across the hiPSC colony with or without ring-barrier. Data are average ± S.D. of 5 cross-sections from the colonies. Significance was determined by one-way ANOVA with Tukey's test (**P < 0.01, *P < 0.05). All experiments were repeated independently at least three times with similar results

To define the relative contributions of mechanical forces on the self-organization of cells, we reconstituted hiPSCs colonies in a ring culture system under culture conditions of pluripotency maintenance. We performed fluorescence staining of phosphorylated myosin light chain (pMLC), which interacts with F-actin bundles to generate contractile force in cultured cells. On day 0, a three-dimensional (3D) reconstruction of the colonies based on staining of F-actin pMLC revealed a multilayered appearance alongside the internal walls inside a confined space made from the ring culture system (Fig. 3A). pMLC colocalized with visibly pronounced F-actin development at the edges of the self-organized hiPSC colony. Quantification of the co-localization of pMLC with F-actin also revealed a peak near the colony edge, further suggesting enriched actomyosin activity at the edge near the barrier of the ring culture system (Fig. 3B). Considering the observed edge retraction upon ablation, this suggests the presence of mechanical tension at the overhanging edges of the self-organized hiPSC colony. Immunostaining of pluripotency markers OCT3/4 and SOX2 revealed loss of OCT3/4 expression in cells at the colony edge, while SOX2 expression was maintained in cells localized throughout the colony (Additional file 1: Fig. S1A). In addition, immunostaining of Ki67, a cell proliferation marker was carried out, and this indicated that cells at the colony edge still had increased proliferation as compared to cells at the colony center (Additional file 1: Fig. S1B). Taken together, these results suggest that the involvement of mechanical tension at the edge of a self-organized hiPSC colony can in turn influence the pluripotency state even under culture conditions that maintain pluripotency.

To better understand this process, we examined the 3D spatial structure of F-actin and pMLC in the colonies cultured in a KSR-based medium without the addition of exogenous supplements. In cultures with a ring-barrier, extended actin filaments assembled into network-like structures, particularly at sites of intercellular connections within a compact cell colony on day 2 (Fig. 3A and Additional file 2: Movie S1). The cells at the colony center formed adherent monolayers of polygonal cells that possessed a defined vertical organization to the actin cytoskeleton. F-actin was highly enriched at the colony edges with clearly visible bundles compared with cells at the colony center. In addition, the cells at the colony center exhibited cortical actin network but lacked visible F-actin bundles. F-actin and pMLC were more enriched closer to the apical side of cells at the edge of the colony, while no enrichment was observed at the center, indicating that such a polarized distribution facilitates the balance of stresses arising from actomyosin contractility. The pMLC staining intensity in F-actin bundles also revealed a peak at the edge near the barrier of the ring culture system (at the location of the ring barrier) (Fig. 3B).

In cultures without a ring-barrier, actin fibers increased in cells within the hiPSC colony through the regulation of pMLC during collective cell migration on day 2 (Fig. 3A and Additional file 3: Movie S2). The multilayered cells at the edge near the barrier of the ring culture system (at the location of the ring barrier) were monolayered by cell migration at the edge of the colony. Actin stress fibers were abundant along the apical and basal sides of the cells in monolayers. They exhibited epithelial-like characteristics with specialized cell–cell junctions anchored by apical bundles of actin filaments. Moreover, actin-based plasma membrane protrusions called lamellipodia and filopodia were concentrated at the leading edge of the colony. Compared with cells at the colony center, cells at the ring barrier and colony edge exhibited pronounced expression of pMLC that interacted with F-actin bundles to generate contractile force. On the apical side, the thick circumferential belt of F-actin was interconnected between the cells and strongly colocalize with pMLC. Both F-actin and pMLC were observed at the same level of staining intensity from the center to the edge of the colony (Fig. 3B).

In cultures where the ring barrier was removed and exposed to HA, the cells within colonies had a markedly round morphology compared to that of untreated cells, confirming an increase in shrinkage of the apical surface of the cells (Fig. 3A and Additional file 4: Movie S3). pMLC strongly colocalized with F-actin at the apical surface of cells at the colony center. However, the actin fibers increased at the colony edge, and a perpendicular alignment of cells and actin fibers with respect to the direction of stretching was observed. In addition, cells displayed relatively uniform staining intensity of both F-actin and pMLC (Fig. 3B). Taken together, these results suggest that the changes of mechanical tension through collective cell migration at the colony edge may trigger the transition of pluripotency state even in the absence of exogenous supplements.

**Fig. 4:**
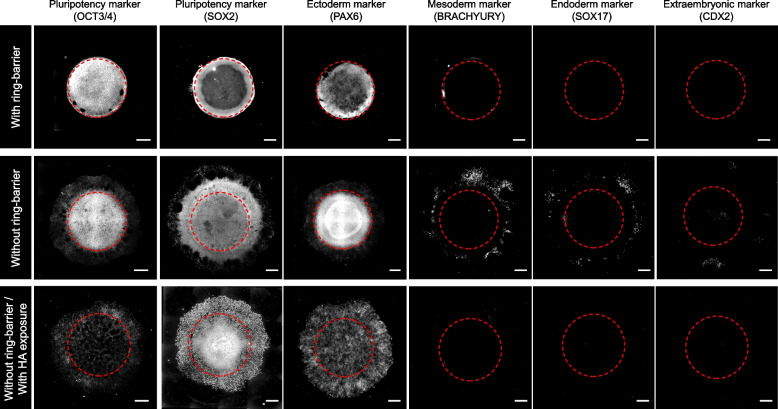
Characterization of spatial self-organization within the hiPSC colony with or without the ring-barrier in the absence of exogenous differentiation factors. Immunofluorescent images of a pluripotency markers (OCT3/4 and SOX2) and differentiation markers including ectoderm (PAX6), mesoderm (BRACHYURY), endoderm (SOX17), and extra-embryonic trophoblast (CDX2). The hiPSC colonies were compared after further exposure to HA in culture conditions without the ring-barrier. Red dashed lines indicate location of the ring culture system. Scale bar, 500 μm. All experiments were repeated independently at least three times with similar results

**Fig. 5:**
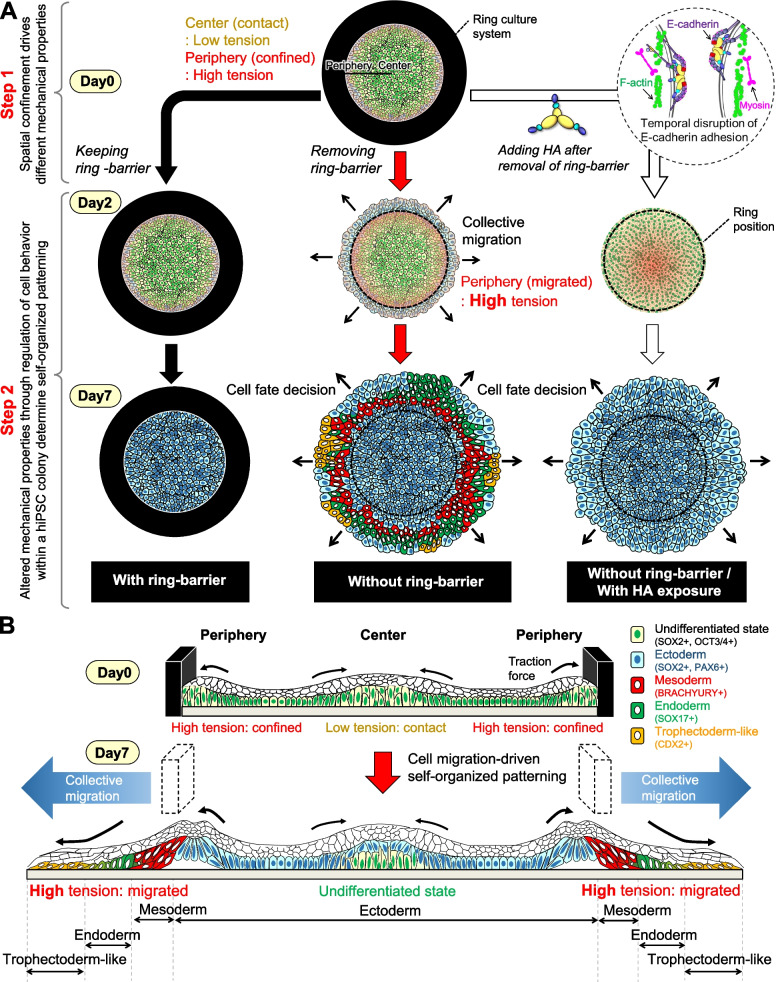
Schematic of our hypothesis on the mechanism by which collective cell migration induced by the removal of ring-barrier induces the spatial self-organization into gastrulation-like phenotype. **A** Comparison of spatial self-organization within the hiPSC colony with or without the ring-barrier in the absence of exogenous differentiation factors. In the first step, hiPSCs self-assemble into a colony primarily through cell–substrate and cell–cell adhesion. In the second step, by removing the ring-barrier, the hiPSC colony spontaneously differentiated into SOX17^+^/BRACHYURY^+^ gastrulation-like structures through collective cell migration without exogenous supplements. The hiPSC colonies were compared after further exposure to HA in culture conditions without the ring-barrier. **B** A detailed description of the spatial self-organization process within the hiPSC colony through collective cell migration in the absence of exogenous differentiation factor

To determine the pluripotency status and lineage commitment within the hiPSC colony, we immunostained the pluripotency markers OCT3/4 and SOX2, ectodermal marker PAX6, definitive endoderm marker SOX17, and mesoderm/primitive streak marker BRACHYURY on day 7. Immunostaining of pluripotency marker OCT3/4 revealed that OCT3/4 expression was maintained localized in cells through the colony in culture with or without a ring-barrier, while loss of OCT3/4 expression was throughout the colony in culture without a ring-barrier through disruption of cadherin-based cell junctions with HA exposure (Fig. 4A). SOX2 is a core transcription factor that is maintained during ectodermal differentiation but downregulated during meso–endodermal differentiation [5]. The SOX2 and PAX6 were expressed throughout the spatially confined hiPSC colony within the ring culture system. However, in cultures without a ring-barrier, the cells at the colony center expressed SOX2 and PAX6, marking the prospective ectoderm. The rings at progressively larger radii expressed BRACHYURY, SOX17, and CDX2, marking the emergence of mesoderm, endoderm, and extra-embryonic trophoblasts, respectively. These findings suggest a two-part mechanism whereby spatially restricted high cell-adhesion tension initiates at the colony edge and then feeds forward to drive collective cell migration that induces meso–endodermal specification to reinforce the gastrulation-like phenotype even in the absence of exogenous differentiation factors (Fig. 5). Accordingly, inhibition of collective cell migration through disruption of cadherin-based cell junctions with HA exposure was restricted to ectodermal differentiation only and did not develop other lineage-differentiated cells.

### Characterization of self-organization of spatial patterning in hiPSCs with or without ring culture systems under the influence of endodermal differentiation condition

**Fig. 6:**
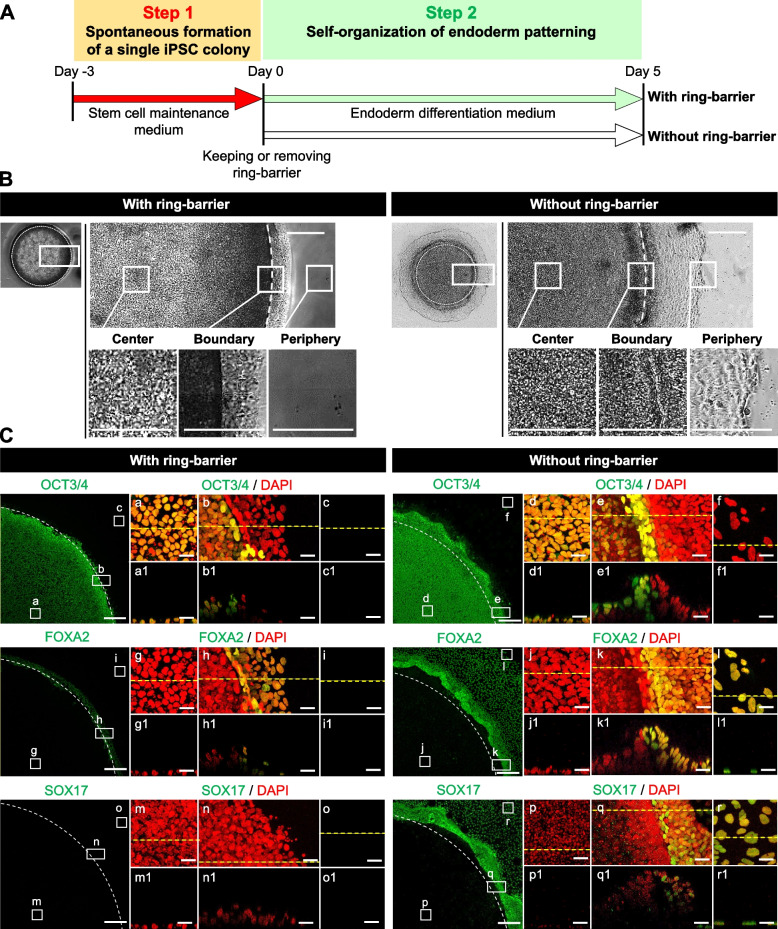
Characterization of spatial self-organization within the hiPSC colony with or without the ring-barrier using the ring culture system under the influence of endodermal differentiation conditions. **A** Experimental design for endodermal differentiation based on the ring culture system. **B** Representative morphologies are shown at different time points during endodermal differentiation within the hiPSC colony with or without the ring-barrier. Scale bar, 500 μm. **C** Representative images in the *XY* plane showing expression of pluripotency (OCT3/4) and endodermal (FOXA2, SOX17) markers. Images are shown as one quarter of a colony. Panels a1–r1 are the tomograms sectioned at the *XZ* plane (yellow dashed lines) in panels a-r. White dashed lines indicate location of the ring culture system. Nuclei were stained with DAPI. Scale bar, 50 μm. All experiments were repeated independently at least three times with similar results

**Fig. 7:**
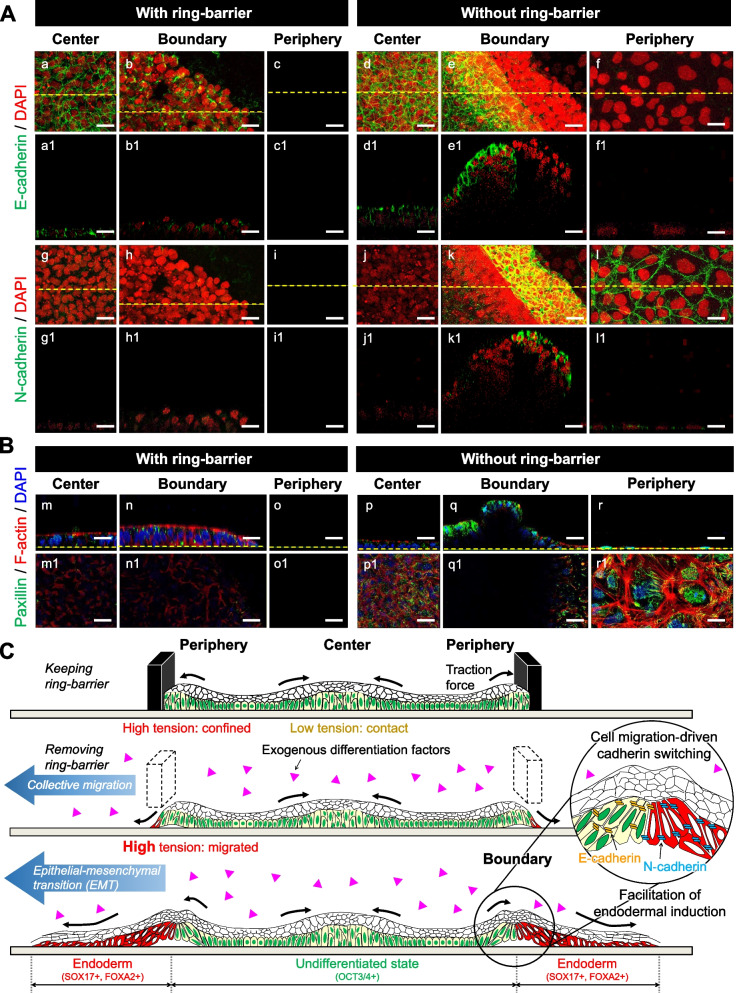
Characterization of spatial self-organization within the hiPSC colony with or without the ring-barrier in the absence of exogenous differentiation factors. **A** Representative images in the *XY* plane showing the expression of E-cadherin and N-cadherin within the hiPSC colony. Panels a1–l1 are the tomograms sectioned at the *XZ* plane (yellow dashed lines) in panels a-l. Nuclei were stained with DAPI. Scale bar, 50 μm. **B** Representative images in the *XY* plane showing the expression of paxillin within the hiPSC colony. Panels m1–r1 are the tomograms sectioned at the *XY* plane (yellow dashed lines) in panels m-r. Nuclei were stained with DAPI. Scale bar, 50 μm. **C** A detailed description of the endodermal differentiation process within the hiPSC colony through collective cell migration. Differences in spatial self-organization within the hiPSC colonies in cultures with and without the ring-barrier removal are shown. All experiments were repeated independently at least three times with similar results

To investigate the effect of collective cell migration on self-organization within the hiPSC colony, we tested the differentiation potential of the cells as they transitioned from undifferentiated to endodermal progenitors by exchanging the endodermal induction media after 3 days of hiPSC colony formation. We performed time-lapse observations of the hiPSC differentiation culture after the removal of the ring-barrier on day 0. The cells at the colony edge collectively migrated towards the newly available free space, resulting in colony patterns that arise primarily through cell migration (Additional file 5: Movie S4). The cells at the colony edge with distinct boundaries began to migrate outwards into free space and remained on the perimeter of the colony to eventually form a band of differentiation. When switching to the endodermal induction media on day 0, in cultures with the ring-barrier, OCT3/4 was still expressed throughout the colony by day 7, while SOX17 and FOXA2 were expressed in a few cells at the colony edge (Fig. 6). In cultures without a ring barrier, however, the cells at the center expressed OCT3/4, whereas the OCT3/4 was completely absent at the colony edge. The SOX17 and FOXA2 were expressed in cells at the colony edge. The above results argue that in cultures without the ring-barrier, cells integrate information from the exogenous differentiation factors to create an endoderm pattern within the colony. We found that in a conventional culture with endodermal differentiation media, the cells were a mixture of dense and relatively dispersed parts (Fig. S2). Interestingly, the OCT3/4 expression was still observed in tightly packed regions with cells, whereas the SOX17 expression was completely absent in the densely packed cells. This result is consistent with current research showing that the cells at the center region within the spatially confined hiPSC colony within the ring culture system retain the expression of OCT3/4. We hypothesized that the cell fate decision in the second step of the differentiation induction depends on EMT due to collective cell migration at the colony edge capable of outward migration. Cell migration in differentiating hPSCs was tightly linked and had been attributed to the process of EMT, which was characterized by reduced E-cadherin and increased N-cadherin expression [4, 13, 14]. To investigate the mechanisms of the effect of cell migration-driven EMT in cultures with and without the ring-barrier under the influence of endodermal differentiation condition, we performed immunostaining for two cell–cell adhesion-associated proteins, E-cadherin and N-cadherin, and a cell-substrate adhesion-associated protein, paxillin at day 7. In cultures without the ring-barrier, E-cadherin expression appeared at the colony center, whereas N-cadherin was expressed at the colony edge (Fig. 7A). In contrast, E-cadherin was expressed at cell–cell contacts within colonies in cultures with the ring-barrier. Cell nuclei at the boundary region were partially overlapped and detached from the substrate. In this regard, the relationship between switching from E-cadherin to N-cadherin was shown for the formation of a boundary separating undifferentiated and differentiated cells by collective cell migration at the colony edge under the influence of endodermal differentiation conditions.

Subsequently, the results of fluorescent staining of paxillin and F-actin showed that actin stress fibers were abundant along basal sides of cells at the colony edge in cultures without the ring-barrier (Fig. 7B). Many of the spots of paxillin, a focal adhesion protein, were distributed at the leading edge with lamellipodia and strong paxillin spots at the basal side. A comparison of the central and peripheral regions of the colony revealed an increase in actin filaments and paxillin spots in cells located at the colony edge. In contrast, in cultures with a ring-barrier, the cells showed reduced actin filaments on the basal side and weak paxillin staining with small, thin adhesions throughout the colony. Collectively, these findings demonstrate that collective cell migration and EMT at the colony edge in cultures with the ring-barrier are required to promote the induction of hiPSC-derived endoderm differentiation (Fig. 7C).

## Discussion

Collective cell migration has long been recognized to be necessary to drive the spatial organization associated with the developmental transition, and we provide compelling evidence that these collective cell migrations influence cell fate specification by modifying cellular response to mechanical forces [4, 28–30]. Here, we developed a ring culture system to induce formation of self-organized hiPSC colony, enabling us to associate a local mechanical condition in colony edge with alteration in pluripotency state, and investigated the role of collective cell migration behaviors in spatial self-organization on hiPSC differentiation. We presented a simple strategy for producing a removable ring-shaped physical barrier to study the effects of collective cell migration in spatially confined hiPSC colonies (Fig. 1). It is made of PDMS, a flexible, transparent and biocompatible material that is gas permeable but barely permeable to water. The ring-barrier contains a magnet that can be inserted into a culture vessel to prevent cells from moving outside the defined area. In addition, the ring-barrier can be removed without damaging the edge of the colony formed on the ring barrier. This action was sufficient to trigger the migration of the cell collectives without the need to add other humoral factors such as growth factors. The schematic representation shown in Fig. 5 summarizes a process involving a two-step cell fate pattern within a hiPSC colony in a ring culture system. The main findings of this study are as follows: First, our study found that ring-barrier geometrically confines the hiPSC colony and spatially presents mechanical stress. In addition, it was shown that spatially confined hiPSC colonies within the ring-barrier alone can trigger the self-organization of hiPSCs to differentiate gastruloid-like structures. After cells initially adhere within the ring-barrier, cell migration and proliferation result in the formation of large, single colonies. During cell colony formation, the cell–substrate and cell–cell adhesion cooperate to organize actomyosin networks and maintain force transmission [31–36]. The ability of hPSCs to form colonies depends on the reorganization of the cytoskeleton, the contraction of actin filaments, cell–cell interactions, and the timely function of the regulatory protein [32, 33]. In cultures with a ring-barrier, the cells at the edge of hiPSC colonies have high growth ability under stem cell maintenance culture, showing distinct cortical actin organization, increased activation of myosin, and strong traction forces between the cells and ECM (Fig. 3). There are physical forces transmitted through the cells that affect the local mechanical properties and, therefore, display remarkable spatial heterogeneity of pluripotency within the same colony, even under culture conditions that maintain pluripotency [34, 35]. When switching to the a KSR-based medium without the addition of exogenous supplements, low OCT3/4 expression in the outer region of spatially confined hiPSC colony coincided with F-actin and pMLC enrichment (Fig. 3). In addition, we found that spatially confined hiPSC colony can differentiate into outer trophectoderm-like, intermediate mesendoderm-like and an inner ectoderm-like cells by inducing collective cell migration after removal of ring-barrier even under culture condition without the addition of exogenous supplements (Fig. 4). The gastrulation-like structures observed in this study are similar to those reported in previous studies of spatial patterns resulting from morphogen gradients [11, 13, 14] and both systems resulted in mesodermal specification occurring near the colony edge. This positional difference of the colony edge from the center has been proposed as a fundamental mechanism for spontaneous symmetry breaking and pattern formation in early embryos [6, 29, 37–41]. Previous studies examining the relationship between cell adhesion tension and gastrulation-like phenotype in hPSCs have shown that cell–cell adhesion mediates the high tension required to control gastrulation cell movements [6, 29]. It has been shown that germ layer separation during gastrulation is driven by other forces in addition to cell–cell adhesion, which reveals that actomyosin-cortex-dependent surface tension is important for sorting mesodermal and ectodermal progenitor cells [38–41]. Ectodermal cells that adhere via the weakest homophilic adhesion exhibit the strongest cell surface tension, but mesodermal cells that adhere via higher homophilic affinity exhibit the weakest cell surface tension. Conversely, inhibition of myosin contractility reduces the tension of ectodermal cells and inhibits sorting from mesodermal cells without inhibiting adhesion. Recent studies have found that the differential presentation of receptor proteins and gradient of humoral factors influence the fate of cells in hPSC colonies, in addition to different mechanical properties depending on the cell’s position in the hPSC colony [5, 35, 42]. It was demonstrated that the cell position-dependent mechanical properties in apical structures and functions within hPSC colonies differently affect cellular response to morphogen inputs, such as BMP and NODAL [5, 9]. Thus, the developed platform in this study provides an engineered tool to study the role of mechanical properties of cells in self-organization and pattern formation processes.

Second, our study found that induction of collective cell migration facilitated meso–endodermal lineage segregation and cell fate decisions within hiPSC colonies. In collective cell migration, cell–cell adhesions require to maintain the adhesion of the migrating group. The actomyosin contractility remains high around the migrating cells at the hPSC colony edge, generating pulling forces [33, 34]. Importantly, the collective cell migration requires suppression of actomyosin at cell–cell contact, and suppression of actomyosin at cell–cell contact results in loss of cohesion of migrating cells at the outermost edge of the colony [29]. The cells form self-organized differentiation patterns in the concentric radial region during collective cell migration, which express germ layer-specific markers, reminiscent of gastrulating embryos. The mechanosensitive focal adhesion proteins FAK and paxillin, play a key role by changing their conformations in response to forces transmitted at the intercellular junctions that trigger signaling events [21, 38]. These results are consistent with studies in hiPSC colonies in which cytoskeletal tension at the colony edge was found to be important for leader cell formation during collective cell migration (Fig. 3). This behavior of contractile cells at the boundary coordinating EMT has been observed in vivo [4, 14]. However, disruption of cadherin-mediated intercellular adhesion invalidates directional collective cell migration, and passive intercellular adhesion tends to move cells uniformly regardless of geometry [4, 28–30]. Accordingly, disruption of E-cadherin-based adherens junctions using HA minimized the effects of force-generating actomyosin cytoskeleton of neighboring cells and subsequently enabled directed collective cell migration (Fig. 3). This resulted in differential patterns within hiPSC colonies owing to cellular self-organization while retaining an ectodermal fate (Fig. 4). Altered mechanical force by disrupting cell–cell adhesion would change force distributions across adhesion receptors and result in both local and global restructuring of the tensionally integrated cytoskeleton network [34]. As a result, cell populations within the hiPSC colonies may acquire edge-like properties when cells lose polarity, suggesting that their gene expression profiles may change. Taken together, these results revealed that biomechanics of collective cell migration during development may be sufficient to alter cell fate even in the absence of exogenous differentiation factors. This allowed us to improve and gives a better understanding of the role of collective cell migration on spatial self-organization within the hiPSC colony.

Furthermore, we found that the spatial organization of confined hiPSC colonies promotes differentiation progression at the colony edge by inducing collective cell migration after the removal of the ring-barrier under the influence of endodermal differentiation condition (Fig. 6A). Differentiating cells at the colony edge underwent a cadherin switch to N-cadherin expression (Fig. 7). The gain of N-cadherin expression and loss of E-cadherin expression, which is associated with the initial stage of EMT required for the specification of definitive endoderm, promotes differentiation to meso–endodermal lineages [13, 15, 21]. We further investigated the distribution and direction of focal adhesion points at different locations of the collectives because much of the cellular traction is known to be transmitted through the site of focal adhesion. The cells at the colony edge showed increased staining of paxillin (Fig. 7). It is likely that inducing collective cell migration at the colony edge by removing the ring-barrier enhanced the mechanical signaling response by the alternation of cell–cell adhesion and cell–substrate adhesion, thus acting synergistically on the input of exogenous differentiation factors (Fig. 7C). However, it is unknown how mechanical forces in collective cell migration driven by leader cells interact with paracrine signaling and exogenous chemical factors. In addition, although no bioengineering strategies have been established to alter the mechanical properties of hiPSC migration, it was found that the mechanical properties of cells migrating at the edge of the colony contribute to the specification of the fate of the lineage. Thus further investigation focusing on chemical and mechanical signal integration during collective cell migration in the process of self-organization of geometrically migrated colonies will be required to enhance endodermal differentiation from hPSCs. These results make it likely that pathways regulate cell lineages and demonstrate that this knowledge can be used profitably to guide stem cell fate toward specific lineages in controlled the differentiation and stabilization of differentiation process for the generation of stem cell therapy products.

## Conclusions

We developed a physical barrier using a removable magnetic ring to induce the formation of a single colony, enabling us to associate colony edges with alterations in pluripotency during the spatial self-organization of hiPSCs. By removal of the ring-barrier, hiPSC colony spontaneously differentiated into an outer trophectoderm-like ring, an inner ectodermal circle, and a ring of meso–endodermal expressing primitive-streak markers in between, and collective cell migration-induced cadherin switching in meso–endodermal lineage segregation in the absence of differentiation-inducing factors. In addition, collective cell migration at the colony edge provides a physical niche that is sufficient to promote endodermal differentiation efficiency within the hiPSC colony. The developed in vitro culture platform can be used to gain insight into the regulatory mechanisms of germ layer formation in migratory behavior control and can be considered a practical approach to regulate the fate and differentiation of stem cells in vitro.

## Methods

### Cell preparation

The hiPSC line, 1383D2, was obtained from the Center for iPS Cell Research and Application, Kyoto University (Kyoto, Japan). Cells were maintained on culture substrates coated with recombinant laminin-511 E8 fragments (iMatrix-511; Nippi, Inc., Tokyo, Japan) in a chemically defined and animal component-free medium (StemFit AK02N; Ajinomoto Co., Inc., Tokyo, Japan) following a previously published protocol [43]. The cells were cultured at a viable cell density of 7.5 × 10^3^ cells/cm^2^ and subcultured every four days using TrypLE Select. For the first 24 h, 10 μM Rho-associated coiled-coil containing protein kinase (ROCK) inhibitor (Y-27632; Wako Pure Chemical Industries, Osaka, Japan) was used to enhance single hiPSC survival. The cells were incubated at 37 °C in a humidified atmosphere containing 5% CO_2_, and the culture medium was replaced daily with fresh medium.

### Experimental design

Figure 1 depicts the experimental design for undifferentiated hiPSC colony formation and induction of lineage differentiation in the ring culture system. For spontaneous and directed differentiation of hiPSCs, cells in the ring culture system were allowed to proceed for another seven days of differentiation with or without the physical ring-barrier. This culture system can easily build an internal culture region and an external cell-free region by establishing a temporal physical barrier on the culture surface using a removable magnetic ring. Removal of this physical barrier provides sufficient free space to trigger collective cell migration.

#### Step 1. Formation of a single hiPSC colony using a ring culture system

For undifferentiated hiPSC colony formation, cells in the ring culture system were cultured in the same medium used to maintain pluripotency. Prior to cell seeding, the culture surface was coated with iMatrix (0.25 μg/cm^2^) for 2 h at 37 °C and rinsed with PBS after 2 h for cellular attachment. To set up a closed culture vessel, a culture plate was placed on a stainless steel plate. The rings were then placed vertically at the bottom of the culture vessel to create a physical barrier. Cells were subsequently seeded inside the ring culture system at 1.0 × 10^5^ cells/cm^2^ density with iMatrix in StemFit AK02N medium supplemented with 10 μM ROCK inhibitor as the pluripotency maintenance medium and then incubated for 30 min at 37 °C with 5% CO_2_ to allow cell attachment to the culture surface. To discard unbound cells, the mesh substrates seeded with cells were transferred to a new culture dish, and the culture was continued using a fresh culture medium. Following initial incubation, the same culture medium containing ROCK inhibitors was added to the cultures for the first 24 h to prevent apoptosis after single-cell dissociation. The culture medium was replaced with a fresh medium without ROCK inhibitors every 24 h for three days.

#### Step 2. Self-organization of cell fate and endodermal differentiation within an hiPSC colony with or without ring-barrier using a ring culture system

For spontaneous differentiation of hiPSC colonies, cells were cultured without exogenous growth factors that favor a particular lineage. The basal differentiation medium for hiPSCs contained Glasgow Minimum Essential Medium (Sigma-Aldrich, St. Louis, MO, USA) supplemented with knockout serum replacement (KSR; Invitrogen, Carlsbad, CA, USA), 1 mM sodium pyruvate, 1 mM l-glutamine, 1% non-essential amino acids, 0.1 mM 2-mercaptoethanol, 50 μg/ml penicillin, and 50 μg/ml streptomycin. On day 0, the medium was replaced with a 10% KSR-containing basal differentiation medium, and cells were cultured for seven days. The medium was changed every day. In some experiments, cells were cultured in a medium containing 60 nM botulinum hemagglutinin (HA) for 24 h to inhibit E-cadherin-based cell–cell adhesion.

The hiPSC colonies were differentiated into definitive endodermal cells using a STEMdiff definitive endoderm kit (Stemcell Technologies, Vancouver, Canada). The culture medium was changed every day for up to seven days.

### Time-lapse live-cell imaging

To monitor the differentiation process of hiPSCs in monolayer culture, we used a phase-contrast time-lapse observation incubator (BioStudio T; Nikon, Tokyo, Japan) equipped with a camera for video imaging through a 4 × objective lens. The chamber within the incubator was maintained at 37 °C with 5% CO_2_.

### Immunofluorescence staining

Immunofluorescence staining was performed as previously described [21]. Briefly, the cells were fixed with 4% paraformaldehyde (Fujifilm Wako Pure Chemical Corporation, Osaka, Japan) for 10 min, rinsed with phosphate-buffered saline (PBS), and permeabilized with 0.5% Triton X-100 in PBS for 5 min. For blocking the attachment of non-specific proteins, the cells were exposed to Block Ace (Dainippon Sumitomo Pharma Co. Ltd., Osaka, Japan) for 90 min at room temperature and then immersed at 4 °C overnight in primary antibody solution. The primary antibodies in PBS with 10% Block Ace. For immunolabeling, the cells were rinsed with Tris-buffered saline and immersed in a secondary antibody solution for 1 h. The primary and secondary antibodies are provided in Additional file 6: Table S1. F-actin and cell nuclei were stained with a rhodamine phalloidin and 4’,6-diamidino-2-phenylindole (both obtained from Thermo Fisher Scientific) in PBS, respectively. The cells were observed using an image analyzer with 10 × and 20 × objective lenses (IN Cell Analyzer 2000; GE Healthcare, Japan) or a confocal laser microscope (Model FV-1000; Olympus, Tokyo, Japan) with a 60 × objective lens and fluorescence excitation at 358, 488, and 594 nm. Quantification of fluorescence intensity in the images was conducted by intensity profile analysis using Image-Pro Plus 7.0 software (Media Cybernetics Inc., Rockville, MD, USA).

### Statistical analysis

All experiments were repeated independently at least three times with similar results, and representative data are shown. Differences between multiple groups were analyzed by one-way analysis of variance (ANOVA) followed by Tukey's post-hoc test. The significance thresholds were **P < 0.01 and *P < 0.05.

## Supplementary Information

**Additional file 1: Fig. S1.** Characterization of spatial self-organization in the hiPSC colony with and without the ring-barrier. Representative immunofluorescent images of pluripotency markers (OCT3/4, SOX2) (A) and proliferation marker (Ki67) (B) within the hiPSC colony cultured in the ring culture system at the end of colony formation (at day 0). Scale bar, 200 μm. **Fig. S2.** Characterization of hiPSC differentiation potential in normal endodermal differentiation culture on day 4. Representative image for cell morphology (A) and immunofluorescent images of a pluripotency marker (OCT3/4) and endodermal marker (SOX17) (B). Nuclei were stained with DAPI. Scale bar, 200 μm.

**Additional file 2: Movie S1.** 3D reconstruction of *Z*-stack of F-actin and pMLC at different positions of the hiPSC colony in culture with the ring-barrier. Full *Z*-stack of the images in Fig. 3. Representative images in the *XY* plane show F-actin and pMLC within the hiPSC colony. Reconstruction of images in the *XZ* plane showing F-actin and pMLC at the location of the yellow dashed line in each *XY* image. The movie follows the colony from bottom to top and from right to left. Scale bar, 50 μm.

**Additional file 3: Movie S2.** 3D reconstruction of Z-stack of F-actin and pMLC at different positions of the hiPSC colony in culture without the ring barrier. Full *Z*-stack of the images in Fig. 3. Representative images in the *XY* plane showing F-actin and pMLC within the hiPSC colony. Reconstruction of images in the *XZ* plane showing F-actin and pMLC at the location of the yellow dashed line in each *XY* image. The movie follows the colony from bottom to top and from right to left. Scale bar, 50 μm.

**Additional file 4: Movie S3.** 3D reconstruction of *Z*-stack of F-actin and pMLC at different positions of the hiPSC colony in culture without the ring-barrier / with HA exposure. Full *Z*-stack of the images in Fig. 3. Representative images in the *XY* plane show F-actin and pMLC within the hiPSC colony exposed to HA. Reconstruction of images in the *XZ* plane showing F-actin and pMLC at the location of the yellow dashed line in each *XY* image. The movie follows the colony from bottom to top and from right to left. Scale bar, 50 μm.

**Additional file 5: Movie S4.** Time-lapse morphological observations from day 0 to day 5 during endodermal differentiation within the hiPSC colony in culture without the ring-barrier. Scale bar, 1 mm.

**Additional file 6: Table S1.** List of primary and secondary antibodies used in staining experiments.

## Data Availability

The datasets used and/or analyzed during the current study are available from the corresponding author on reasonable request.
