# Supplementary material for: An in vitro culture platform for studying the effect of collective cell migration on spatial self-organization within induced pluripotent stem cell colonies
Source: J Biol Eng. 2023 Mar 30;17:25. doi: 10.1186/s13036-023-00341-z (PMC10064534; doi:10.1186/s13036-023-00341-z)
Supplement: Supplementary file 1 — Additional file 1: Fig. S1. Characterization of spatial self-organization in the hiPSC colony with and without the ring-barrier. Representative immunofluorescent images of pluripotency markers (OCT3/4, SOX2) (A) and proliferation marker (Ki67) (B) within the hiPSC colony cultured in the ring culture system at the end of colony formation (at day 0). Scale bar, 200 μm. Fig. S2. Characterization of hiPSC differentiation potential in normal endodermal differentiation culture on day 4. Representative image for cell morphology (A) and immunofluorescent images of a pluripotency marker (OCT3/4) and endodermal marker (SOX17) (B). Nuclei were stained with DAPI. Scale bar, 200 μm. [file 13036_2023_341_MOESM1_ESM.docx]

**
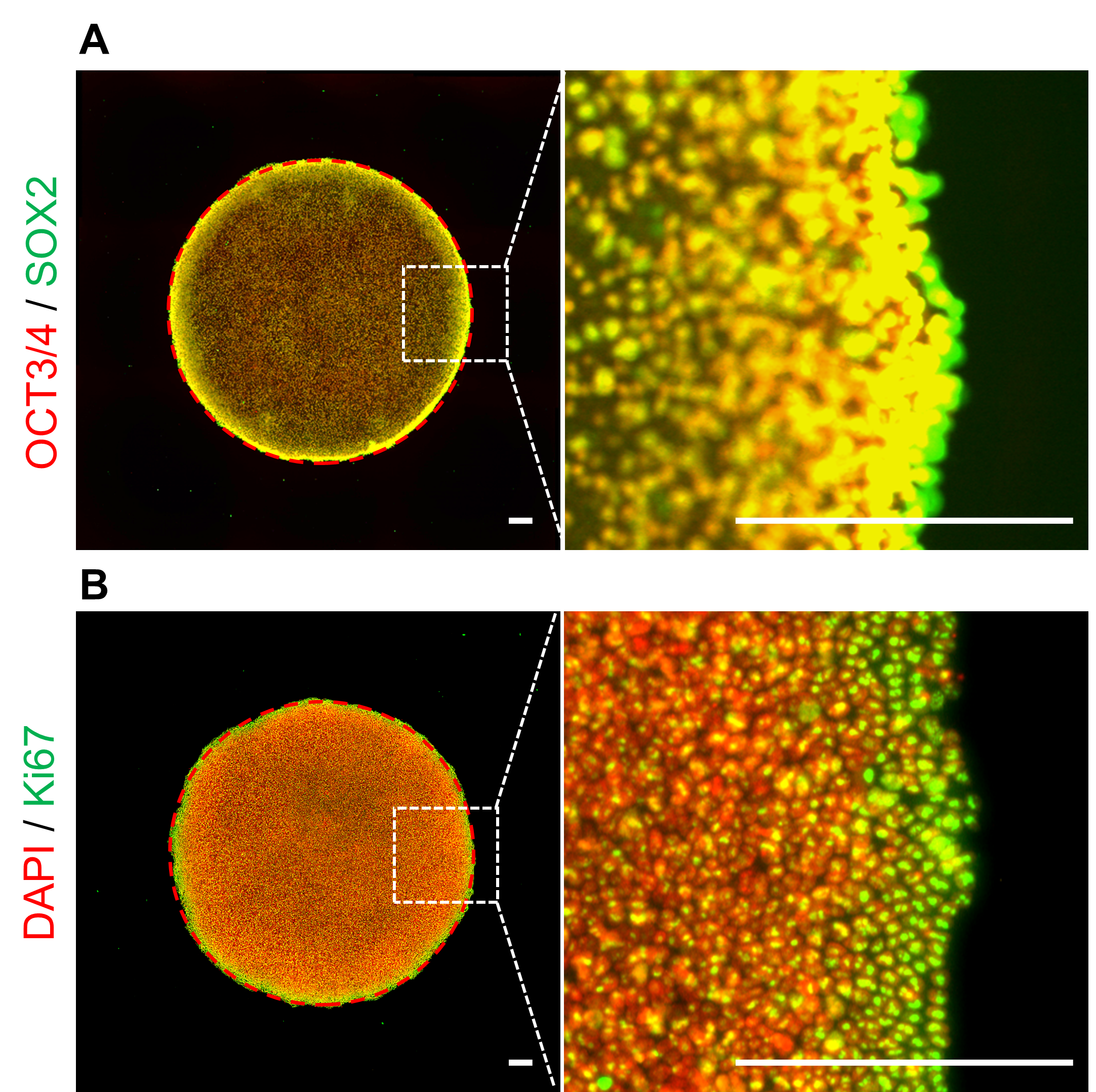
**

**Fig. S1.** Characterization of spatial self-organization in the hiPSC colony with and without the ring-barrier. Representative immunofluorescent images of pluripotency markers (OCT3/4, SOX2) (A) and proliferation marker (Ki67) (B) within the hiPSC colony cultured in the ring culture system at the end of colony formation (at day 0). Scale bar, 200 μm.

**
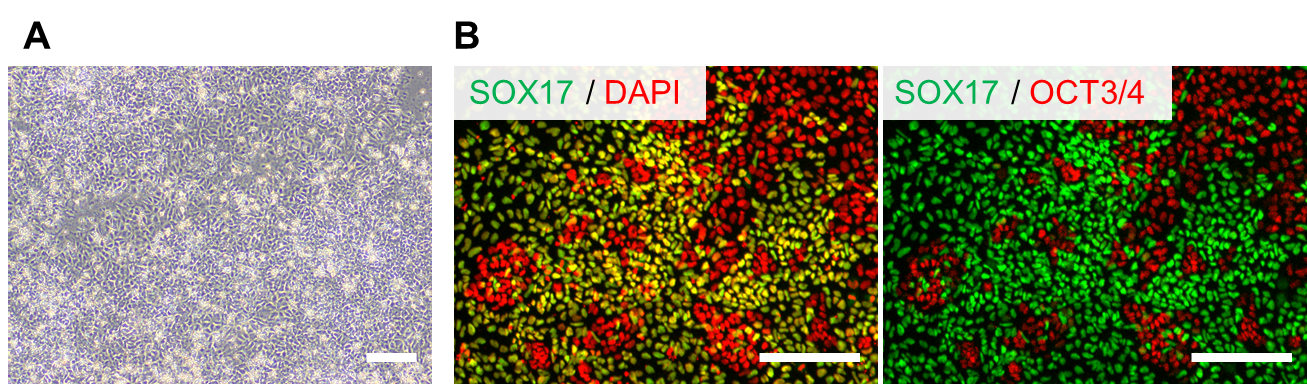
**

**Fig. S2.** Characterization of hiPSC differentiation potential in normal endodermal differentiation culture on day 4. Representative image for cell morphology (A) and immunofluorescent images of a pluripotency marker (OCT3/4) and endodermal marker (SOX17) (B). Nuclei were stained with DAPI. Scale bar, 200 μm.
