# Supplementary material for: An in vitro culture platform for studying the effect of collective cell migration on spatial self-organization within induced pluripotent stem cell colonies
Source: J Biol Eng. 2023 Mar 30;17:25. doi: 10.1186/s13036-023-00341-z (PMC10064534; doi:10.1186/s13036-023-00341-z)
Supplement: Supplementary file 6 — Additional file 6: Table S1. List of primary and secondary antibodies used in staining experiments. [file 13036_2023_341_MOESM6_ESM.docx]

**Table S1**. List of primary and secondary antibodies used in staining experiments.

| **Antibodies** | **Source** | **Identifier** |
| --- | --- | --- |
| Anti-phospho-myosin light chain (pMLC) antibody | Cell Signaling Technology Inc. | 3675S |
| Anti-E-cadherin antibody | Cell Signaling Technology Inc. | 3195S |
| Anti-N-cadherin antibody | BD Biosciences | 610920 |
| Anti-OCT3/4 antibody | Santa Cruz Biotechnology | sc-5279 |
| Anti-SOX2 antibody | Cell Signaling Technology Inc. | 3579S |
| Anti-BRACHYURY antibody | Cell Signaling Technology Inc. | 81694S |
| Anti-SOX17 antibody | Cell Signaling Technology Inc. | 81778S |
| Anti-FOXA2 antibody | Santa Cruz Biotechnology | sc-374376 |
| Anti-CDX2 antibody | Cell Signaling Technology Inc. | 12306S |
| Anti-Ki67 antibody | Abcam | ab16667 |
| Alexa Fluor 488-conjugated goat anti-mouse IgG antibody | ThermoFisher Scientific | A-11001 |
| Alexa Fluor 594-conjugated goat anti-mouse IgG antibody | ThermoFisher Scientific | A-11005 |
| Alexa Fluor 488-conjugated goat anti-rabbit IgG antibody | ThermoFisher Scientific | A-11008 |
| Alexa Fluor 594-conjugated goat anti-rabbit IgG antibody | ThermoFisher Scientific | A-11012 |
